# 9-{[4-(Dimethyl­amino)­benz­yl]amino}-5-(3,4,5-trimeth­oxy­phen­yl)-5,5a,8a,9-tetra­hydro­furo[3′,4′:6,7]naphtho­[2,3-*d*][1,3]dioxol-6(8*H*)-one

**DOI:** 10.1107/S1600536811019234

**Published:** 2011-05-28

**Authors:** Yan Li, Huo Wang, Hong Chen, Li-Ting Chen, Jing Liu

**Affiliations:** a The Pharmacy Department of the General Hospital, of the Chinese People’s Armed Police Force, Beijing 100039, People’s Republic of China; b The Affiliated Hospital of the Medical College of, the Chinese People’s Armed Police Forces, Tianjin 300162, People’s Republic of China; c Tianjin Key Laboratory for Biomarkers of Occupational, and Environmental Hazards, Tianjin 300162, People’s Republic of China; d Room of Pharmacognosy, Medical College of Chinese People’s Armed Police Forces, Tianjin 300162, People’s Republic of China

## Abstract

In the title compound, C_31_H_34_N_2_O_7_, the fused tetra­hydro­furan and six-membered rings each display an envelope conformation. The dihedral angles between the benzene ring of the benzo[*d*][1,3]dioxole and the other two benzene rings are 89.68 (3) and 63.38 (2)°. In the crystal, weak inter­molecular C—H⋯O hydrogen bonds link the mol­ecules.

## Related literature

For details of the synthesis and biological activity of podophyllotoxin (systematic name (10*R*,11*R*,15*R*,16*R*)-16-hy­droxy-10-(3,4,5-trimeth­oxy­phen­yl)-4,6,13-trioxatetra­cyclo­[7.7.0.03,7.011,15]hexa­deca-1,3(7),8-trien-12-one) derivatives, see: Yu *et al.* (2008 ▶); Zhao *et al.* (2009 ▶); Lu *et al.* (2010 ▶). For related structures, see: Zhang *et al.* (1994 ▶); Feng *et al.* (2008 ▶); Zuo *et al.* (2009 ▶).
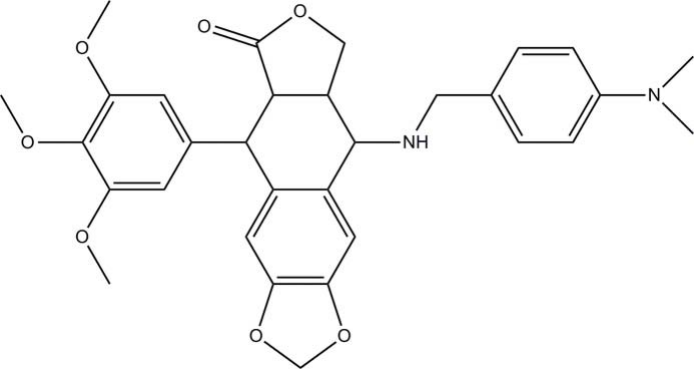


## Experimental

### 

#### Crystal data

C_31_H_34_N_2_O_7_
*M* *_r_* = 546.60
Monoclinic, 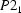

*a* = 10.188 (2) Å
*b* = 11.530 (3) Å
*c* = 11.691 (3) Å
β = 96.192 (4)°
*V* = 1365.4 (6) Å^3^
*Z* = 2
Mo *K*α radiation
μ = 0.09 mm^−1^
*T* = 113 K
0.20 × 0.18 × 0.12 mm

#### Data collection

Rigaku Saturn CCD area-detector diffractometer
Absorption correction: multi-scan (*CrystalClear*; Rigaku/MSC, 2007 ▶) *T*_min_ = 0.981, *T*_max_ = 0.989
17611 measured reflections
6335 independent reflections
4878 reflections with *I* > 2σ(*I*)
*R*_int_ = 0.034

#### Refinement

*R*[*F* ^2^ > 2σ(*F* ^2^)] = 0.028
*wR*(*F* ^2^) = 0.061
*S* = 1.02
6335 reflections
370 parameters
2 restraints
H atoms treated by a mixture of independent and constrained refinement
Δρ_max_ = 0.16 e Å^−3^
Δρ_min_ = −0.21 e Å^−3^

### 

Data collection: *CrystalClear* (Rigaku/MSC, 2007 ▶); cell refinement: *CrystalClear*; data reduction: *CrystalClear*; program(s) used to solve structure: *SHELXS97* (Sheldrick, 2008 ▶); program(s) used to refine structure: *SHELXL97* (Sheldrick, 2008 ▶); molecular graphics: *SHELXTL* (Sheldrick, 2008 ▶); software used to prepare material for publication: *SHELXL97*.

## Supplementary Material

Crystal structure: contains datablocks I, global. DOI: 10.1107/S1600536811019234/cv5091sup1.cif

Structure factors: contains datablocks I. DOI: 10.1107/S1600536811019234/cv5091Isup2.hkl

Supplementary material file. DOI: 10.1107/S1600536811019234/cv5091Isup3.cml

Additional supplementary materials:  crystallographic information; 3D view; checkCIF report

## Figures and Tables

**Table 1:** Hydrogen-bond geometry (Å, °)

*D*—H⋯*A*	*D*—H	H⋯*A*	*D*⋯*A*	*D*—H⋯*A*
C1—H1*B*⋯O6^i^	0.99	2.38	3.2904 (16)	153
C21—H21*A*⋯O6^ii^	0.98	2.51	3.3662 (18)	145
C22—H22*B*⋯O3^iii^	0.98	2.54	3.4909 (18)	162
C29—H29*C*⋯O1^iv^	0.98	2.49	3.3017 (18)	140

Symmetry codes: (i) 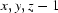; (ii) 
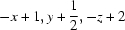
; (iii) 
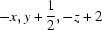
; (iv) 
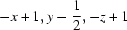
.

**Fig. 1.**
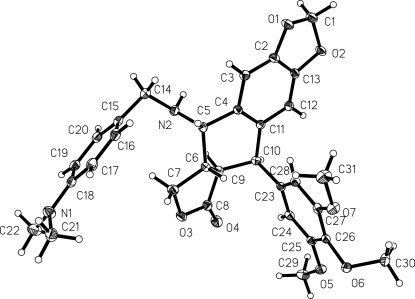
View of the title compound, with displacement ellipsoids drawn at the 40% probability level.

## supplementary crystallographic information

### Comment

Podophyllotoxin and its derivatives are well known as substances with anti-cancer activity. In our group, we synthesize different kinds of Podophyllotoxin compounds in the search for new derivatives with improved bioactivities (Lu *et al.*, 2010; Yu *et al.*, 2008; Zhao *et al.*, 2009). In this paper, we present the crystal structure of the title compound (I).

In (I) (Fig. 1),the  bond lengths and angles are normal and in a good agreement with those reported previously for related compounds (Feng *et al.*, 2008; Zhang *et al.*, 1994; Zuo *et al.*, 2009). The tetrahydrofuran ring (C6—C9/O3) and the six-membered ring (C4—C6/C9—C11) fused to it both display envelope conformations. The dihedral angles between the benzene ring (C2—C4/C11—C13) of the benzo[*d*]-[1,3]dioxole and the other two benzene ring (C23—C88 and C15—C20) are 89.68 (3) and 63.38 (2) °, respectively.

Weak intermolecular C—H···O interactions (Table 1) stabilize the crystal packing.

### Experimental

The target compound was synthesized in two steps. 4-(Dimethylamino)benzaldehyde, 4β-amino podophyllotoxin, two drops of acetic acid in 95% ethanol was stirred for 6 h. Appropriate amount of NaBH_4_ was added into the reaction mixture to stirred for 1 h at 273 K. Then add 5% HCl to end off the reaction, the reaction mixture was concentrated *in vacuo*. Add saturated NaHCO_3_ to adjust PH>7. The reaction mixture was extracted with CH_2_Cl_2_ and dried over MgSO_4_ and concentrated *in vacuo*. The residue was resolved in a methanol solution and slow evaporation over two weeks at room temperature gave transparent crystals suitable for X-ray analysis.

### Refinement

C-bound H atoms were found on difference maps, but placed in idealized positions with C—H = 0.95–1.00 Å, and refined as riding, with *U*_iso_(H) = 1.2*U*_eq_(C) for aryl and methylene H atoms and 1.5*U*_eq_(C) for the methyl H atoms. N-bound H atoms were located on a difference map and isotropically refined. In the absence of any significant anomalous scatterers in the molecule, the 2920 Friedel pairs were merged before the final refinement.

### Crystal data

**Table:**

C_31_H_34_N_2_O_7_	*F*(000) = 580
*M**_r_* = 546.60	*D*_x_ = 1.329 Mg m^−^^3^
Monoclinic, *P*2_1_	Mo *K*α radiation, λ = 0.71073 Å
Hall symbol: P 2yb	Cell parameters from 4484 reflections
*a* = 10.188 (2) Å	θ = 1.8–28.1°
*b* = 11.530 (3) Å	µ = 0.09 mm^−^^1^
*c* = 11.691 (3) Å	*T* = 113 K
β = 96.192 (4)°	Prism, colourless
*V* = 1365.4 (6) Å^3^	0.20 × 0.18 × 0.12 mm
*Z* = 2	

### Data collection

**Table:**

Rigaku Saturn CCD area-detector diffractometer	6335 independent reflections
Radiation source: rotating anode	4878 reflections with *I* > 2σ(*I*)
multilayer	*R*_int_ = 0.034
Detector resolution: 14.63 pixels mm^-1^	θ_max_ = 27.8°, θ_min_ = 1.8°
ω and φ scans	*h* = −13→13
Absorption correction: multi-scan (*CrystalClear*; Rigaku/MSC, 2007)	*k* = −15→15
*T*_min_ = 0.981, *T*_max_ = 0.989	*l* = −15→15
17611 measured reflections	

### Refinement

**Table:**

Refinement on *F*^2^	Primary atom site location: structure-invariant direct methods
Least-squares matrix: full	Secondary atom site location: difference Fourier map
*R*[*F*^2^ > 2σ(*F*^2^)] = 0.028	Hydrogen site location: inferred from neighbouring sites
*wR*(*F*^2^) = 0.061	H atoms treated by a mixture of independent and constrained refinement
*S* = 1.02	*w* = 1/[σ^2^(*F*_o_^2^) + (0.024*P*)^2^] where *P* = (*F*_o_^2^ + 2*F*_c_^2^)/3
6335 reflections	(Δ/σ)_max_ = 0.005
370 parameters	Δρ_max_ = 0.16 e Å^−^^3^
2 restraints	Δρ_min_ = −0.21 e Å^−^^3^

### Special details

**Table:**

Geometry. All e.s.d.'s (except the e.s.d. in the dihedral angle between two l.s. planes) are estimated using the full covariance matrix. The cell e.s.d.'s are taken into account individually in the estimation of e.s.d.'s in distances, angles and torsion angles; correlations between e.s.d.'s in cell parameters are only used when they are defined by crystal symmetry. An approximate (isotropic) treatment of cell e.s.d.'s is used for estimating e.s.d.'s involving l.s. planes.
Refinement. Refinement of *F*^2^ against ALL reflections. The weighted *R*-factor *wR* and goodness of fit *S* are based on *F*^2^, conventional *R*-factors *R* are based on *F*, with *F* set to zero for negative *F*^2^. The threshold expression of *F*^2^ > σ(*F*^2^) is used only for calculating *R*-factors(gt) *etc*. and is not relevant to the choice of reflections for refinement. *R*-factors based on *F*^2^ are statistically about twice as large as those based on *F*, and *R*- factors based on ALL data will be even larger.

### Fractional atomic coordinates and isotropic or equivalent isotropic displacement parameters (Å^2^)

**Table:**

	*x*	*y*	*z*	*U*_iso_*/*U*_eq_	
O1	0.52128 (9)	0.59140 (8)	0.21947 (8)	0.0240 (2)	
O2	0.52819 (9)	0.39712 (8)	0.27006 (7)	0.0233 (2)	
O3	0.14528 (9)	0.64023 (8)	0.82671 (7)	0.0235 (2)	
O4	0.14154 (9)	0.44619 (9)	0.83314 (8)	0.0282 (2)	
O5	0.53808 (8)	0.28789 (8)	0.99527 (8)	0.0213 (2)	
O6	0.74780 (8)	0.42130 (8)	0.99096 (7)	0.0214 (2)	
O7	0.77467 (8)	0.56748 (8)	0.81392 (8)	0.0235 (2)	
N1	−0.02700 (11)	1.09298 (11)	0.83706 (10)	0.0263 (3)	
N2	0.14526 (11)	0.72761 (10)	0.48482 (9)	0.0210 (3)	
C1	0.53397 (14)	0.47595 (12)	0.17577 (12)	0.0244 (3)	
H1A	0.4615	0.4596	0.1145	0.029*	
H1B	0.6191	0.4675	0.1430	0.029*	
C2	0.46171 (12)	0.57629 (12)	0.31872 (11)	0.0181 (3)	
C3	0.40468 (12)	0.65809 (12)	0.38261 (10)	0.0187 (3)	
H3	0.3992	0.7370	0.3591	0.022*	
C4	0.35439 (12)	0.62146 (11)	0.48439 (10)	0.0158 (3)	
C5	0.27834 (12)	0.71177 (11)	0.54717 (10)	0.0167 (3)	
H5	0.3268	0.7873	0.5483	0.020*	
C6	0.27250 (13)	0.67110 (11)	0.67159 (10)	0.0166 (3)	
H6	0.3641	0.6717	0.7122	0.020*	
C7	0.18142 (14)	0.73142 (12)	0.74730 (11)	0.0219 (3)	
H7A	0.2274	0.7963	0.7902	0.026*	
H7B	0.1021	0.7620	0.7007	0.026*	
C8	0.16817 (12)	0.53318 (12)	0.78400 (11)	0.0202 (3)	
C9	0.22116 (12)	0.54719 (12)	0.66907 (11)	0.0164 (3)	
H9	0.1437	0.5439	0.6088	0.020*	
C10	0.32353 (12)	0.46168 (11)	0.63313 (10)	0.0160 (3)	
H10	0.2802	0.3843	0.6197	0.019*	
C11	0.36598 (12)	0.50503 (12)	0.51885 (10)	0.0160 (3)	
C12	0.42322 (12)	0.42254 (12)	0.45006 (11)	0.0179 (3)	
H12	0.4305	0.3433	0.4723	0.022*	
C13	0.46778 (12)	0.46049 (12)	0.35037 (11)	0.0177 (3)	
C14	0.11461 (15)	0.84675 (13)	0.44324 (12)	0.0265 (3)	
H14A	0.0420	0.8443	0.3797	0.032*	
H14B	0.1932	0.8807	0.4130	0.032*	
C15	0.07453 (13)	0.92169 (12)	0.53913 (11)	0.0218 (3)	
C16	0.15867 (13)	1.00166 (12)	0.59750 (12)	0.0234 (3)	
H16	0.2414	1.0168	0.5701	0.028*	
C17	0.12671 (13)	1.06049 (13)	0.69451 (12)	0.0240 (3)	
H17	0.1869	1.1151	0.7314	0.029*	
C18	0.00521 (12)	1.03947 (12)	0.73842 (11)	0.0204 (3)	
C19	−0.08234 (13)	0.96224 (12)	0.67618 (11)	0.0217 (3)	
H19	−0.1670	0.9491	0.7008	0.026*	
C20	−0.04779 (13)	0.90515 (12)	0.58008 (11)	0.0216 (3)	
H20	−0.1091	0.8530	0.5406	0.026*	
C21	0.07587 (14)	1.15428 (13)	0.91019 (12)	0.0274 (3)	
H21A	0.1551	1.1057	0.9220	0.041*	
H21B	0.0443	1.1713	0.9847	0.041*	
H21C	0.0972	1.2270	0.8730	0.041*	
C22	−0.14462 (14)	1.05611 (15)	0.88653 (12)	0.0321 (4)	
H22A	−0.2228	1.0745	0.8333	0.048*	
H22B	−0.1499	1.0966	0.9596	0.048*	
H22C	−0.1407	0.9723	0.9002	0.048*	
C23	0.44248 (12)	0.44741 (11)	0.72446 (10)	0.0160 (3)	
C24	0.43331 (13)	0.37052 (11)	0.81531 (11)	0.0175 (3)	
H24	0.3558	0.3252	0.8181	0.021*	
C25	0.53765 (12)	0.36013 (11)	0.90182 (11)	0.0164 (3)	
C26	0.65162 (12)	0.42673 (12)	0.89914 (10)	0.0163 (3)	
C27	0.66085 (12)	0.50269 (11)	0.80689 (11)	0.0176 (3)	
C28	0.55777 (12)	0.51155 (11)	0.71925 (11)	0.0168 (3)	
H28	0.5658	0.5614	0.6557	0.020*	
C29	0.41589 (13)	0.23341 (13)	1.01374 (11)	0.0260 (3)	
H29A	0.3478	0.2928	1.0183	0.039*	
H29B	0.4272	0.1894	1.0858	0.039*	
H29C	0.3890	0.1807	0.9497	0.039*	
C30	0.87104 (13)	0.37267 (14)	0.96535 (13)	0.0309 (4)	
H30A	0.8542	0.3019	0.9197	0.046*	
H30B	0.9254	0.3539	1.0373	0.046*	
H30C	0.9176	0.4290	0.9216	0.046*	
C31	0.78655 (14)	0.65140 (14)	0.72551 (12)	0.0301 (4)	
H31A	0.7865	0.6119	0.6513	0.045*	
H31B	0.8693	0.6944	0.7426	0.045*	
H31C	0.7120	0.7054	0.7221	0.045*	
H2	0.1326 (14)	0.6802 (12)	0.4235 (10)	0.033 (4)*	

### Atomic displacement parameters (Å^2^)

**Table:**

	*U*^11^	*U*^22^	*U*^33^	*U*^12^	*U*^13^	*U*^23^
O1	0.0322 (6)	0.0216 (5)	0.0202 (5)	0.0008 (4)	0.0114 (4)	−0.0022 (4)
O2	0.0307 (5)	0.0213 (5)	0.0195 (5)	0.0047 (4)	0.0098 (4)	−0.0025 (4)
O3	0.0282 (5)	0.0229 (5)	0.0212 (5)	0.0033 (4)	0.0106 (4)	0.0016 (4)
O4	0.0291 (6)	0.0260 (6)	0.0319 (6)	−0.0013 (5)	0.0136 (5)	0.0057 (5)
O5	0.0240 (5)	0.0214 (5)	0.0187 (5)	−0.0029 (4)	0.0036 (4)	0.0054 (4)
O6	0.0164 (5)	0.0301 (6)	0.0177 (5)	0.0007 (4)	0.0017 (4)	0.0006 (4)
O7	0.0188 (5)	0.0261 (6)	0.0253 (5)	−0.0074 (4)	0.0013 (4)	0.0065 (4)
N1	0.0224 (6)	0.0318 (7)	0.0243 (6)	−0.0011 (5)	0.0013 (5)	−0.0075 (6)
N2	0.0236 (6)	0.0206 (6)	0.0179 (6)	0.0047 (5)	−0.0019 (5)	−0.0031 (5)
C1	0.0325 (8)	0.0221 (8)	0.0198 (7)	0.0017 (7)	0.0087 (6)	−0.0015 (6)
C2	0.0170 (6)	0.0225 (8)	0.0153 (6)	−0.0025 (6)	0.0033 (5)	−0.0005 (6)
C3	0.0218 (7)	0.0157 (7)	0.0185 (7)	−0.0005 (6)	0.0024 (6)	0.0003 (6)
C4	0.0149 (6)	0.0174 (7)	0.0151 (6)	−0.0003 (5)	0.0012 (5)	−0.0014 (5)
C5	0.0166 (6)	0.0155 (7)	0.0181 (7)	−0.0001 (6)	0.0026 (5)	−0.0017 (6)
C6	0.0172 (6)	0.0172 (7)	0.0157 (6)	0.0002 (6)	0.0035 (5)	−0.0017 (6)
C7	0.0276 (8)	0.0190 (7)	0.0203 (7)	−0.0006 (6)	0.0077 (6)	0.0033 (6)
C8	0.0162 (6)	0.0223 (8)	0.0225 (7)	0.0003 (6)	0.0042 (5)	0.0018 (6)
C9	0.0145 (6)	0.0187 (7)	0.0160 (6)	−0.0017 (6)	0.0018 (5)	−0.0010 (6)
C10	0.0176 (7)	0.0148 (7)	0.0156 (6)	−0.0020 (6)	0.0022 (5)	−0.0002 (5)
C11	0.0135 (6)	0.0194 (7)	0.0149 (6)	−0.0008 (6)	0.0003 (5)	−0.0016 (6)
C12	0.0182 (7)	0.0158 (7)	0.0197 (7)	0.0007 (6)	0.0014 (5)	0.0001 (6)
C13	0.0158 (6)	0.0205 (7)	0.0165 (7)	0.0026 (6)	0.0009 (5)	−0.0048 (6)
C14	0.0296 (8)	0.0280 (8)	0.0215 (7)	0.0096 (7)	0.0008 (6)	0.0042 (6)
C15	0.0252 (7)	0.0199 (7)	0.0202 (7)	0.0073 (6)	0.0018 (6)	0.0051 (6)
C16	0.0211 (7)	0.0220 (8)	0.0278 (8)	0.0037 (6)	0.0055 (6)	0.0080 (6)
C17	0.0215 (7)	0.0202 (8)	0.0296 (8)	−0.0006 (6)	0.0000 (6)	0.0023 (6)
C18	0.0200 (7)	0.0182 (7)	0.0224 (7)	0.0044 (6)	−0.0006 (5)	0.0023 (6)
C19	0.0173 (7)	0.0243 (8)	0.0230 (7)	0.0019 (6)	0.0007 (6)	0.0047 (6)
C20	0.0222 (7)	0.0198 (7)	0.0214 (7)	0.0025 (6)	−0.0041 (6)	0.0010 (6)
C21	0.0271 (8)	0.0246 (8)	0.0292 (8)	0.0025 (7)	−0.0030 (6)	−0.0054 (7)
C22	0.0289 (8)	0.0447 (10)	0.0230 (7)	−0.0014 (8)	0.0040 (6)	−0.0040 (7)
C23	0.0185 (6)	0.0152 (7)	0.0147 (6)	0.0017 (5)	0.0039 (5)	−0.0012 (5)
C24	0.0185 (7)	0.0154 (7)	0.0193 (7)	−0.0021 (5)	0.0059 (5)	−0.0012 (6)
C25	0.0217 (7)	0.0138 (7)	0.0146 (6)	0.0014 (6)	0.0067 (5)	0.0020 (5)
C26	0.0160 (6)	0.0181 (7)	0.0148 (6)	0.0022 (5)	0.0017 (5)	−0.0026 (6)
C27	0.0161 (6)	0.0164 (7)	0.0209 (7)	−0.0006 (5)	0.0052 (5)	−0.0013 (6)
C28	0.0204 (7)	0.0145 (7)	0.0160 (6)	−0.0002 (5)	0.0048 (5)	0.0028 (6)
C29	0.0313 (8)	0.0253 (8)	0.0225 (7)	−0.0079 (7)	0.0088 (6)	0.0050 (6)
C30	0.0242 (8)	0.0364 (9)	0.0311 (8)	0.0124 (7)	−0.0007 (6)	−0.0026 (7)
C31	0.0271 (8)	0.0318 (9)	0.0316 (8)	−0.0096 (7)	0.0041 (7)	0.0113 (7)

### Geometric parameters (Å, °)

**Table:**

O1—C2	1.3773 (15)	C11—C12	1.4118 (18)
O1—C1	1.4367 (17)	C12—C13	1.3676 (17)
O2—C13	1.3852 (15)	C12—H12	0.9500
O2—C1	1.4349 (16)	C14—C15	1.5063 (19)
O3—C8	1.3610 (16)	C14—H14A	0.9900
O3—C7	1.4753 (15)	C14—H14B	0.9900
O4—C8	1.2014 (16)	C15—C16	1.387 (2)
O5—C25	1.3734 (15)	C15—C20	1.3955 (18)
O5—C29	1.4314 (15)	C16—C17	1.3901 (19)
O6—C26	1.3746 (15)	C16—H16	0.9500
O6—C30	1.4359 (16)	C17—C18	1.4113 (18)
O7—C27	1.3744 (15)	C17—H17	0.9500
O7—C31	1.4305 (16)	C18—C19	1.4064 (19)
N1—C18	1.3784 (16)	C19—C20	1.3802 (18)
N1—C22	1.4494 (17)	C19—H19	0.9500
N1—C21	1.4615 (17)	C20—H20	0.9500
N2—C14	1.4792 (18)	C21—H21A	0.9800
N2—C5	1.4798 (16)	C21—H21B	0.9800
N2—H2	0.900 (9)	C21—H21C	0.9800
C1—H1A	0.9900	C22—H22A	0.9800
C1—H1B	0.9900	C22—H22B	0.9800
C2—C3	1.3707 (18)	C22—H22C	0.9800
C2—C13	1.3851 (19)	C23—C24	1.3945 (17)
C3—C4	1.4100 (17)	C23—C28	1.3949 (18)
C3—H3	0.9500	C24—C25	1.3912 (17)
C4—C11	1.4030 (18)	C24—H24	0.9500
C4—C5	1.5317 (18)	C25—C26	1.3954 (18)
C5—C6	1.5356 (17)	C26—C27	1.4004 (17)
C5—H5	1.0000	C27—C28	1.3896 (17)
C6—C7	1.5184 (17)	C28—H28	0.9500
C6—C9	1.5206 (18)	C29—H29A	0.9800
C6—H6	1.0000	C29—H29B	0.9800
C7—H7A	0.9900	C29—H29C	0.9800
C7—H7B	0.9900	C30—H30A	0.9800
C8—C9	1.5098 (18)	C30—H30B	0.9800
C9—C10	1.5268 (17)	C30—H30C	0.9800
C9—H9	1.0000	C31—H31A	0.9800
C10—C11	1.5319 (16)	C31—H31B	0.9800
C10—C23	1.5351 (17)	C31—H31C	0.9800
C10—H10	1.0000		
			
C2—O1—C1	104.30 (10)	C15—C14—H14A	109.5
C13—O2—C1	104.16 (10)	N2—C14—H14B	109.5
C8—O3—C7	110.56 (9)	C15—C14—H14B	109.5
C25—O5—C29	117.25 (10)	H14A—C14—H14B	108.1
C26—O6—C30	114.88 (9)	C16—C15—C20	116.70 (12)
C27—O7—C31	117.45 (10)	C16—C15—C14	123.00 (13)
C18—N1—C22	118.96 (12)	C20—C15—C14	120.05 (13)
C18—N1—C21	119.05 (11)	C15—C16—C17	122.62 (12)
C22—N1—C21	118.69 (11)	C15—C16—H16	118.7
C14—N2—C5	115.23 (11)	C17—C16—H16	118.7
C14—N2—H2	107.1 (10)	C16—C17—C18	120.32 (13)
C5—N2—H2	111.3 (10)	C16—C17—H17	119.8
O2—C1—O1	107.52 (10)	C18—C17—H17	119.8
O2—C1—H1A	110.2	N1—C18—C19	121.42 (12)
O1—C1—H1A	110.2	N1—C18—C17	121.65 (12)
O2—C1—H1B	110.2	C19—C18—C17	116.92 (12)
O1—C1—H1B	110.2	C20—C19—C18	121.33 (12)
H1A—C1—H1B	108.5	C20—C19—H19	119.3
C3—C2—O1	128.56 (12)	C18—C19—H19	119.3
C3—C2—C13	121.80 (12)	C19—C20—C15	121.99 (13)
O1—C2—C13	109.63 (11)	C19—C20—H20	119.0
C2—C3—C4	117.89 (12)	C15—C20—H20	119.0
C2—C3—H3	121.1	N1—C21—H21A	109.5
C4—C3—H3	121.1	N1—C21—H21B	109.5
C11—C4—C3	120.13 (12)	H21A—C21—H21B	109.5
C11—C4—C5	122.96 (11)	N1—C21—H21C	109.5
C3—C4—C5	116.71 (12)	H21A—C21—H21C	109.5
N2—C5—C4	109.38 (10)	H21B—C21—H21C	109.5
N2—C5—C6	112.16 (10)	N1—C22—H22A	109.5
C4—C5—C6	108.64 (10)	N1—C22—H22B	109.5
N2—C5—H5	108.9	H22A—C22—H22B	109.5
C4—C5—H5	108.9	N1—C22—H22C	109.5
C6—C5—H5	108.9	H22A—C22—H22C	109.5
C7—C6—C9	102.19 (10)	H22B—C22—H22C	109.5
C7—C6—C5	120.06 (11)	C24—C23—C28	119.75 (12)
C9—C6—C5	108.48 (10)	C24—C23—C10	119.03 (11)
C7—C6—H6	108.5	C28—C23—C10	121.21 (11)
C9—C6—H6	108.5	C25—C24—C23	119.99 (12)
C5—C6—H6	108.5	C25—C24—H24	120.0
O3—C7—C6	104.48 (10)	C23—C24—H24	120.0
O3—C7—H7A	110.9	O5—C25—C24	124.55 (12)
C6—C7—H7A	110.9	O5—C25—C26	114.86 (11)
O3—C7—H7B	110.9	C24—C25—C26	120.58 (12)
C6—C7—H7B	110.9	O6—C26—C25	118.83 (11)
H7A—C7—H7B	108.9	O6—C26—C27	121.91 (12)
O4—C8—O3	121.68 (11)	C25—C26—C27	119.12 (11)
O4—C8—C9	129.48 (13)	O7—C27—C28	124.74 (12)
O3—C8—C9	108.74 (11)	O7—C27—C26	114.84 (11)
C8—C9—C6	103.80 (10)	C28—C27—C26	120.38 (11)
C8—C9—C10	119.75 (11)	C27—C28—C23	120.12 (12)
C6—C9—C10	111.61 (10)	C27—C28—H28	119.9
C8—C9—H9	107.0	C23—C28—H28	119.9
C6—C9—H9	107.0	O5—C29—H29A	109.5
C10—C9—H9	107.0	O5—C29—H29B	109.5
C9—C10—C11	107.14 (10)	H29A—C29—H29B	109.5
C9—C10—C23	112.91 (10)	O5—C29—H29C	109.5
C11—C10—C23	111.45 (10)	H29A—C29—H29C	109.5
C9—C10—H10	108.4	H29B—C29—H29C	109.5
C11—C10—H10	108.4	O6—C30—H30A	109.5
C23—C10—H10	108.4	O6—C30—H30B	109.5
C4—C11—C12	120.54 (11)	H30A—C30—H30B	109.5
C4—C11—C10	122.67 (11)	O6—C30—H30C	109.5
C12—C11—C10	116.74 (12)	H30A—C30—H30C	109.5
C13—C12—C11	117.74 (12)	H30B—C30—H30C	109.5
C13—C12—H12	121.1	O7—C31—H31A	109.5
C11—C12—H12	121.1	O7—C31—H31B	109.5
C12—C13—O2	128.42 (12)	H31A—C31—H31B	109.5
C12—C13—C2	121.79 (12)	O7—C31—H31C	109.5
O2—C13—C2	109.74 (11)	H31A—C31—H31C	109.5
N2—C14—C15	110.65 (11)	H31B—C31—H31C	109.5
N2—C14—H14A	109.5		
			
C13—O2—C1—O1	20.33 (13)	C1—O2—C13—C2	−11.56 (14)
C2—O1—C1—O2	−21.44 (14)	C3—C2—C13—C12	−3.4 (2)
C1—O1—C2—C3	−166.58 (13)	O1—C2—C13—C12	175.82 (11)
C1—O1—C2—C13	14.31 (14)	C3—C2—C13—O2	179.03 (11)
O1—C2—C3—C4	−177.49 (12)	O1—C2—C13—O2	−1.79 (15)
C13—C2—C3—C4	1.52 (19)	C5—N2—C14—C15	−80.58 (14)
C2—C3—C4—C11	1.34 (19)	N2—C14—C15—C16	102.22 (16)
C2—C3—C4—C5	−173.71 (11)	N2—C14—C15—C20	−71.91 (16)
C14—N2—C5—C4	−120.42 (12)	C20—C15—C16—C17	2.2 (2)
C14—N2—C5—C6	118.97 (12)	C14—C15—C16—C17	−172.16 (13)
C11—C4—C5—N2	−98.96 (14)	C15—C16—C17—C18	0.6 (2)
C3—C4—C5—N2	75.94 (14)	C22—N1—C18—C19	9.1 (2)
C11—C4—C5—C6	23.77 (16)	C21—N1—C18—C19	168.32 (13)
C3—C4—C5—C6	−161.34 (11)	C22—N1—C18—C17	−171.60 (13)
N2—C5—C6—C7	−47.32 (16)	C21—N1—C18—C17	−12.38 (19)
C4—C5—C6—C7	−168.35 (11)	C16—C17—C18—N1	177.37 (12)
N2—C5—C6—C9	69.46 (13)	C16—C17—C18—C19	−3.3 (2)
C4—C5—C6—C9	−51.57 (13)	N1—C18—C19—C20	−177.35 (12)
C8—O3—C7—C6	−19.07 (13)	C17—C18—C19—C20	3.3 (2)
C9—C6—C7—O3	30.09 (13)	C18—C19—C20—C15	−0.6 (2)
C5—C6—C7—O3	150.07 (11)	C16—C15—C20—C19	−2.2 (2)
C7—O3—C8—O4	−177.51 (12)	C14—C15—C20—C19	172.34 (12)
C7—O3—C8—C9	−0.70 (14)	C9—C10—C23—C24	83.46 (14)
O4—C8—C9—C6	−163.42 (13)	C11—C10—C23—C24	−155.89 (11)
O3—C8—C9—C6	20.10 (13)	C9—C10—C23—C28	−95.20 (13)
O4—C8—C9—C10	−38.2 (2)	C11—C10—C23—C28	25.45 (16)
O3—C8—C9—C10	145.35 (11)	C28—C23—C24—C25	1.60 (18)
C7—C6—C9—C8	−30.27 (13)	C10—C23—C24—C25	−177.08 (11)
C5—C6—C9—C8	−158.04 (10)	C29—O5—C25—C24	−10.00 (18)
C7—C6—C9—C10	−160.58 (10)	C29—O5—C25—C26	169.00 (11)
C5—C6—C9—C10	71.65 (13)	C23—C24—C25—O5	179.42 (12)
C8—C9—C10—C11	−175.07 (11)	C23—C24—C25—C26	0.46 (18)
C6—C9—C10—C11	−53.61 (13)	C30—O6—C26—C25	114.96 (13)
C8—C9—C10—C23	−51.99 (15)	C30—O6—C26—C27	−69.35 (16)
C6—C9—C10—C23	69.47 (13)	O5—C25—C26—O6	−4.51 (17)
C3—C4—C11—C12	−2.49 (19)	C24—C25—C26—O6	174.54 (11)
C5—C4—C11—C12	172.24 (11)	O5—C25—C26—C27	179.68 (11)
C3—C4—C11—C10	174.96 (11)	C24—C25—C26—C27	−1.27 (18)
C5—C4—C11—C10	−10.31 (19)	C31—O7—C27—C28	1.12 (18)
C9—C10—C11—C4	23.65 (16)	C31—O7—C27—C26	−176.55 (11)
C23—C10—C11—C4	−100.33 (14)	O6—C26—C27—O7	2.12 (17)
C9—C10—C11—C12	−158.81 (11)	C25—C26—C27—O7	177.80 (11)
C23—C10—C11—C12	77.21 (14)	O6—C26—C27—C28	−175.65 (11)
C4—C11—C12—C13	0.75 (18)	C25—C26—C27—C28	0.02 (18)
C10—C11—C12—C13	−176.85 (11)	O7—C27—C28—C23	−175.51 (12)
C11—C12—C13—O2	179.26 (12)	C26—C27—C28—C23	2.04 (19)
C11—C12—C13—C2	2.13 (19)	C24—C23—C28—C27	−2.84 (18)
C1—O2—C13—C12	171.03 (13)	C10—C23—C28—C27	175.81 (11)

### Hydrogen-bond geometry (Å, °)

**Table:**

*D*—H···*A*	*D*—H	H···*A*	*D*···*A*	*D*—H···*A*
C1—H1A···O5^i^	0.99	2.59	3.0292 (18)	107
C1—H1B···O6^i^	0.99	2.38	3.2904 (16)	153
C21—H21A···O6^ii^	0.98	2.51	3.3662 (18)	145
C22—H22B···O3^iii^	0.98	2.54	3.4909 (18)	162
C29—H29C···O1^iv^	0.98	2.49	3.3017 (18)	140

Symmetry codes: (i) *x*, *y*, *z*−1; (ii) −*x*+1, *y*+1/2, −*z*+2; (iii) −*x*, *y*+1/2, −*z*+2; (iv) −*x*+1, *y*−1/2, −*z*+1.
